# Non-Invasive Monitoring during Caesarean Delivery: Prevalence of Hypotension and Impact on the Newborn

**DOI:** 10.3390/jcm12237295

**Published:** 2023-11-24

**Authors:** Francesco Vasile, Luigi La Via, Paolo Murabito, Stefano Tigano, Federica Merola, Tiziana Nicosia, Giuseppe De Masi, Andrea Bruni, Eugenio Garofalo, Filippo Sanfilippo

**Affiliations:** 1 Department of Anesthesia and Intensive Care, University Hospital Policlinico “G. Rodolico-San Marco”, 95123 Catania, Italy; frankie.vas@hotmail.it (F.V.); paolomurabito@gmail.com (P.M.); filipposanfi@yahoo.it (F.S.); 2 School of Anesthesia and Intensive Care, University Hospital Policlinico “G. Rodolico-San Marco”, 95123 Catania, Italy; stefanotigano.st@gmail.com (S.T.); merolafede@gmail.com (F.M.);; 3 Department of Anesthesia and Intensive Care, Azienda Ospedaliera “Santa Maria”, 05100 Terni, Italy; g.demasi@aospterni.it; 4 School of Anesthesia and Intensive Care, University “Magna Graecia”, 88100 Catanzaro, Italy; andreabruni87@gmail.com (A.B.); eugenio.garofalo@gmail.com (E.G.); 5 Department of General Surgery and Medical—Surgical Specialties, Section of Anesthesia and Intensive Care, University of Catania, 95123 Catania, Italy

**Keywords:** caesarean section, maternal hypotension, neonatal well-being, hemodynamic monitoring, blood pressure

## Abstract

Background: The aim of our study was to investigate the prevalence of perioperative hypotension after spinal anesthesia for cesarean section using non-invasive continuous hemodynamic monitoring and its correlation with neonatal well-being. Methods: We included 145 patients. Spinal anesthesia was performed with a combination of hyperbaric bupivacaine 0.5% (according to a weight/height scheme) and fentanyl 20 μg. Hypotension was defined as a mean arterial pressure (MAP) < 65 mmHg or <60 mmHg. We also evaluated the impact of hypotension on neonatal well-being. Results: Perioperative maternal hypotension occurred in 54.5% of cases considering a MAP < 65 mmHg and in 42.1% with the more conservative cut-off (<60 mmHg). Severe neonatal acidosis occurred in 1.4% of neonates, while an Apgar score ≥ 9 was observed in 95.9% at 1 min and 100% at 5 min. Conclusions: Continuous non-invasive hemodynamic monitoring allowed an early detection of maternal hypotension leading to a prompt treatment with satisfactory results considering neonatal well-being.

## 1. Introduction

Caesarean section (CS) represents one of the most common surgical interventions performed worldwide; it is a low-risk intervention with maternal mortality rated around 0.1–0.2% [1]. Spinal anesthesia (SA) is the first choice for CS due to the ease of the technique and its rapid onset with a low incidence of severe perioperative complications [2]. However, maternal hypotension is not uncommon [3,4,5], with an incidence ranging between 7.4% and 74.1% [6]. Hypotension usually occurs in the first few minutes after SA and it is related to sympathetic preganglionic nerve fiber blockade [7]. The severity and duration of hypotension may cause not only maternal symptoms (such as nausea, vomiting and dyspnea), but also fetal complications [8]. Nausea and vomiting are significantly more frequent during SA for CS than during non-obstetric surgery. In fact, acute hypotension reduces cerebral perfusion, induces transient brainstem ischemia and activates the vomiting center [3]. Transient cerebral hypoxia may occur, as studies using near-infrared spectroscopy show that hypotension is accompanied by a significant decrease in maternal regional cerebral blood volume, cerebral oxygen saturation and oxygenation [9]. This is consistent with the observation that supplemental oxygen may relieve this nausea. Therefore, acute sympathetic blockade may cause unopposed vagal action and subsequent hyperactivity in the gastrointestinal tract [10]. Dizziness and decreased levels of consciousness and dyspnea may follow severe and prolonged maternal hypotension but are uncommon when blood pressure is treated promptly. Several studies have reported an association between hypotension and fetal complications and low APGAR scores [11,12,13,14,15]. In fact, neonates of women with spinal-induced hypotension present with significant acidosis [11,16], and hypotension of more than a 2 min duration is associated with a significant increase in umbilical venous oxypurines and lipid peroxides, suggestive of ischemia–reperfusion injury [15]. Moreover, the duration of hypotension may be more important than its severity. A transient ≥ 30% decrease in blood pressure did not affect neonatal Apgar scores, the incidence of meconium-stained amniotic fluid or the need for oxygen therapy in the neonate [17]. Hypotension for less than 2 min did not affect neonatal neurobehavioral outcomes [11], whereas maternal hypotension exceeding 4 min was associated with neurobehavioral changes at 4–7 days of life [18]. Prevention and early treatment of maternal hypotension represent a very important target for obstetric anesthesiologists, but there is no single approach in this setting [6,19,20]. In fact, some studies have been recently published on the effects of different drug and fluid administration strategies aimed at treating intraoperative hypotension on improving maternal and fetal outcomes [14,21,22]. In this context, intraoperative non-invasive monitoring systems lead to advanced hemodynamic parameter acquisition without the insertion of an arterial catheter line [23]. This information gathered continuously allows for the early detection of intraoperative maternal hypotension, leading to a prompt management with fluids or vasoconstrictors [24,25]. This study is aimed at verifying whether a proactive approach guided by non-invasive hemodynamic monitoring leads to lower prevalence of maternal intraoperative hypotension.

## 2. Materials and Methods

### 2.1. Study Design

This prospective observational cohort study was conducted in two centers, the University Hospital Policlinico-Vittorio Emanuele “G. Rodolico” in Catania and the “Santa Maria Hospital” in Terni, in accordance with the Declaration of Helsinki. The protocol was approved by the local Ethics Committee (Approval Code: NCT03755271). The study followed the STROBE statement and the STROBE checklist used is provided in the Supplementary Materials section.

### 2.2. Population

The inclusion criteria considered were informed consent (acquired during preoperative anesthesiological evaluation), elective CS performed under SA, age between 18 and 40 years old, American Society of Anesthesiologists (ASA) physical status II and positive preoperative postural change test (PCT) result. Exclusion criteria were refusal to participate, twin pregnancy, contraindication to SA and known cardiovascular diseases suggesting the intraoperative use of invasive arterial blood pressure monitoring. The PCT was performed preoperatively to identify patients at higher risk for perioperative hypotension. The test consists of the evaluation of changes in heart rate (HR) according to postural changes. The HR was monitored in supine position, then in left-side decubitus and finally back to supine position. The test was considered positive if the HR increased by more than 10% from basal conditions [26].

### 2.3. Study Operational Protocol

All patients were admitted to the operating room and monitored with ECG, non-invasive blood pressure and oxygen saturation (SpO2). A peripheral venous line of 18 gauges was inserted. Before performing SA, ClearSight/EV1000™ (Edwards Lifesciences^®^, Irvine, CA, USA) for continuous advanced hemodynamic monitoring was positioned. Apart from providing blood pressure values, ClearSight allows for the monitoring of the cardiac index (CI), stroke volume index (SVI) and stroke volume variation (SVV). We collected data on patient demographics, preoperative comorbidities and intraoperative administration of intravenous fluids and vasoconstrictors. Data on advanced hemodynamic monitoring were collected at seven different pre-specified time points (T0–T6): T0 in supine position, T1 in left lateral position, T2 while performing SA with spinal injection, T3 two minutes after SA, T4 at surgical incision, T5 at uterine incision and T6 during fetal extraction. SA was performed in the left lateral position using a 25-gauge Whitacre needle inserted in the L3–L4 or L4–L5 spaces, with the neuraxial administration of 0.5% hyperbaric bupivacaine (5 mg/mL) and 20 μg of fentanyl. The dosage of bupivacaine was chosen according to a pre-established weight- and height-adjusted scheme [22] (Supplementary Materials S1). Just before performing SA in the left lateral decubitus, a 10 mL/kg fluid challenge with ringer acetate was started (first 500 mL was infused using a pressurized bag and then the remainder with an infusion pump system). Once SA was performed, the patient was returned into supine position while the uterus’s bimanual displacement and the 15° left-side tilting of the surgical table were maintained until the surgical field was draped. Our standardized hypotension treatment protocol entailed vasoconstrictor administration (etilefrine boluses in aliquots of 1–5 mg) for a decrease in maternal MAP < 60 mmHg or decrease in CI < 2.5 L/min/m^2^. Uterotonic agent according to a predefined therapeutic scheme was administered after T6 (last time point of data collection). Apgar scores at 1 and 5 min and umbilical cord pH were recorded after delivery.

### 2.4. Outcomes

In this selected population of pregnant women with increased risk of hypotension (positive PCT) undergoing SA for elective CS, our primary endpoint was to determine the impact of maternal hypotension on neonatal well-being. The analysis of hypotension was performed according two different cut-offs of mean arterial pressure (MAP): <65 mmHg or <60 mmHg. Neonatal well-being was assessed according to the pH measured at the umbilical arterial cord blood and the Apgar scores evaluated at 1 and 5 min.

### 2.5. Statistical Analysis

Statistical analysis was performed focusing on MAP and neonatal pH values, considering two definitions of hypotension as the primary endpoint: MAP < 65 mmHg or MAP < 60 mmHg and vasopressor administration. We also recorded the values for SVI, CI and HR. The occurrence of perioperative hypotension episodes was analyzed in terms of absolute frequency in the study population. We also assessed hypotension episode occurrence at time point intervals (T0–T6). Apgar scores and neonatal blood gas test results from the umbilical cord were collected as well. Kolmogorov–Smirnov was preliminarily performed on hemodynamic data to evaluate statistical normal distribution; subsequently, the Friedman test was used, confirming a significant asymmetrical data distribution (*p* < 0.05). Four different statistical tests (Shapiro–Wilk, Anderson–Darling, Lilliefors, Jarque–Bera) were performed to verify the neonatal pH values’ distribution of two groups obtained considering the two maternal MAP cut-offs. In both cases, we found an asymmetrical distribution, and a non-parametric statistical test (Mann–Whitney) was performed to compare groups.

## 3. Results

A total of 145 consecutive term pregnant women with a positive PCT test were enrolled in this study. All included patients were classified as ASA physical status II, with a mean age of 33 years (±5.2), a mean weight of 74.9 kg (±10.4) and a mean height of 163.4 cm (±4.7).

### 3.1. Hypotension Analysis

Table 1 shows the results of maternal hypotension according to the two pre-specified criteria at the different time points. An overall prevalence of 54.5% was detected when a higher cut-off was used, while considering a cut-off of 60 mmHg decreased its prevalence to 42.1%. Among the different study time points, we found the highest rate of hypotension at T4 (skin incision), with a prevalence of a MAP < 60 mmHg and vasopressor administration of 15.2%, while 33.8% of the population had a MAP between 65 mmHg and 60 mmHg. On the contrary, at T2, only 0.7% of the population suffered from maternal hypotension, and no one had a borderline MAP.

### 3.2. Fluid, Vasoconstrictor and Hemodynamic Variables

The mean value of crystalloids administered during the study period was 750 mL. Table 2 shows the number of episodes of maternal hypotension with a MAP < 60 mmHg and the relative amount of etilefrine administered at the different time points. Mean values of hemodynamic variables are shown in the Supplementary Materials S1. In particular, a dose of 2 milligrams was administered 45 times considering the whole study period, while doses of 3 milligrams, 4 milligrams and 5 milligrams were administered 7, 3 and 6 times, respectively.

### 3.3. Umbilical Cord Blood pH and Well-Being

The newborn data relative to Apgar scores at 1 and 5 min are shown in Table 3.

**Figure 1:**
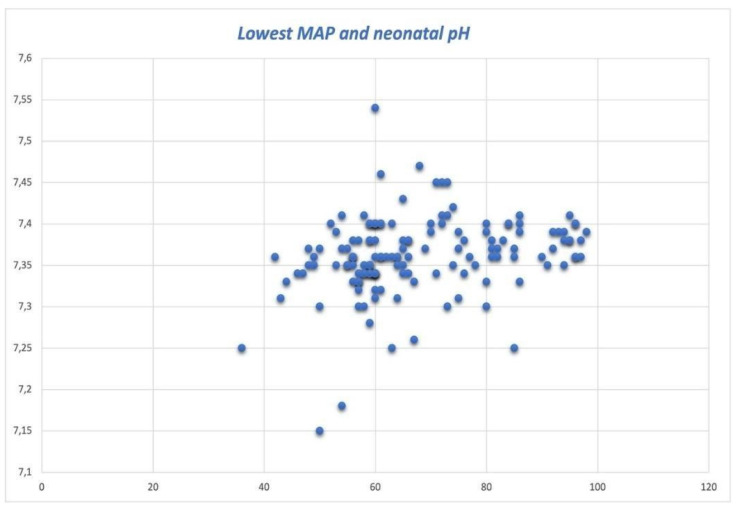
pH value distribution and lowest maternal mean arterial pressure (MAP) values.

Apgar scores below 9 were observed in only 4.1% at 1 min and none at the 5 min evaluation. The mean value of the umbilical arterial cord pH was 7.36 (±0.05). The graphic distribution of the lowest maternal MAP and umbilical arterial cord blood pH is shown in Figure 1 as a scattered plot.

**Figure 2:**
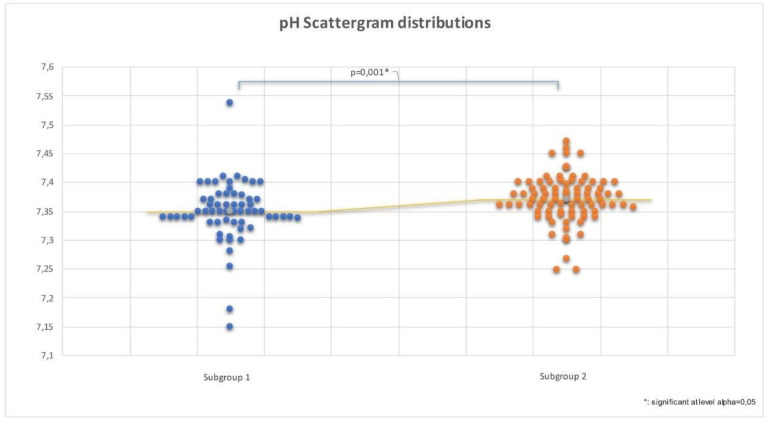
Neonatal pH scattergram distributions of two groups according to the lowest maternal mean arterial pressure (MAP). On the left (blue), the pH values correspond to the group experiencing the lowest maternal MAP below 60 mmHg. On the right (orange), pH values correspond to the group experiencing the lowest maternal MAP above 60 mmHg.

Although significant (*p* < 0.0001), we found that the correlation between the lowest maternal MAP and umbilical pH was weak (rs = 0.332). We analyzed the umbilical cord pH according to the lowest recorded blood pressure by dividing the values into two groups: MAP < 60 mmHg (labeled as Subgroup1, requiring pharmacological intervention) or MAP > 60 mmHg (labeled as Subgroup2). The median pH values recorded were significantly different in the subgroups (7.347 Subgroup1 vs. 7.371 Subgroup2, *p* < 0.0001). Figure 2 shows scattergrams of the pH distribution according to the lowest MAP recorded.

Fetal acidosis (pH < 7.20) occurred only in two cases (1.38%), both in the group with a MAP < 60 mmHg.

## 4. Discussion

In our cohort of pregnant women undergoing CS with a positive PCT result, we found a clinically important prevalence of at least one episode of maternal hypotension. The definition of perioperative hypotension represents a challenge. In the past, the most common definitions of hypotension used in research studies were either “<80% baseline” or “<100 mmHg OR <80% baseline” [27]. A survey conducted in the UK found that most consultant obstetric anesthesiologists used a threshold of either 100 or 90 mmHg [28]. However, the SAP is a less important variable than mean arterial pressure (MAP) as a determinant of organ perfusion. In fact, methods used to measure blood pressure in routine clinical practice have recently started to include MAP, and a value < 65 mmHg in an adult population is now the most widespread cut-off in patients undergoing surgery and in critically ill patients [29,30]. Using a MAP < 65 mmHg as a criterion to define maternal hypotension, in our study, we found that over half of the cohort (54.5%) experienced at least one episode. Treating maternal hypotension as it occurs involves a variety of possible strategies. Recommendations published in 2018 [3] describe that intravenous crystalloid pre-loading has very limited effectiveness at reducing the incidence or severity of hypotension [31], and it is no longer recommended [32,33]. Crystalloid co-loading may be more effective at decreasing hypotension and vasopressor requirements than pre-loading [34] or no fluid therapy at all [35]. Although a meta-analysis suggested no benefit compared to pre-loading [36], a more recent analysis suggested a moderate additional benefit on top of vasopressor prophylaxis, provided that a sufficient volume was infused under pressure during the first 5–10 min after SA [37]. Regarding the type of fluid to infuse, colloid pre-load seems to be more effective than crystalloid pre-load for the prevention of hypotension [31,38]. Moreover, in a recent Cochrane review [31], in order to compare crystalloid with colloid fluid therapy, fewer women experienced hypotension in the colloid group compared with the crystalloid group, but there were no clear differences between groups for maternal hypertension requiring intervention, maternal bradycardia, nausea and/or vomiting, neonatal acidosis or Apgar scores of less than 8 at five minutes. Other measures to prevent or treat hypotension and hemodynamic instability include methods to reduce inferior vena cava compression and venous pooling in the legs, like left uterine displacement with a recommended angle of 15° [39,40]. This angle of table tilt is associated with a higher maternal SAP and cardiac output and lower doses of infused phenylephrine than the unmodified supine position [41], but require improved support and devices for security; however, it can be used during the period of preparation before surgery. Manual displacement of the uterus may be better than left lateral tilt at reducing hypotension at CS [42], but it is difficult to sustain during surgery. Leg compression has been shown to be more effective than no leg compression in preventing hypotension, although a high level of heterogeneity suggests that its effectiveness may depend on the type and intensity of compression used (bandages, inflatable boots or antithromboembolic stockings) [31,43]. In this study, we decided to avoid pharmacological treatment for patients with 65 < MAP < 60 mmHg. Considering this restrictive cut-off, we found a prevalence of at least one episode of maternal hypotension of around 42%. Hypotension episodes mostly occurred in the early phases of the surgical procedure, mainly in T4 (skin incision), followed by T5 (uterine incision). Indeed, we administered a mean amount of almost 750 mL of crystalloids, a value that was 25% lower than suggested by recent recommendations (1000 mL) [6]. Moreover, we did not administer any pre-emptive vasopressors before the SA. Nonetheless, our results well compare with those described in the literature, where an incidence of maternal hypotension has been described in up to 74.1% of the population [27]. Considering neonatal safety aspects, the mean umbilical cord blood pH was 7.36, and we found a significant difference between groups when separating values of pH according to the lowest maternal MAP (<60 and >60 mmHg). However, the median pH values observed were 7.35 and 7.37, a difference that is not clinically meaningful. Indeed, although maternal MAP and neonatal pH showed changes in the same direction, in our cohort of patients, their correlation was weak. It is likely that the most important factor in the development of neonatal metabolic acidosis could be the duration of maternal perioperative hypotension rather than the absolute value of MAP and/or the use of vasoconstrictors. Also, the Apgar scores confirmed neonatal well-being with a score below 9 observed only in 4.1% at 1 min and none at 5 min evaluation. The adequate intraoperative monitoring for term pregnant women undergoing CS with SA is still matter of debate. In fact, recent studies investigated the use of non-invasive hemodynamic monitoring with the implementation of hypotension prediction index software (Acumen IQ) [44,45,46]. These studies showed the effectiveness of a proactive protocol based on an algorithm able to predict intraoperative hypotension [47,48,49]. Future research might explore the efficacy and accuracy of this approach in this particular patient’s population. Moreover, the assessment of fluid responsiveness by the echographic evaluation of the inferior vena cava might be useful in order to optimize the volume status in the perioperative period. However, the subcostal approach is not usually available during term pregnancy. In this case, a transhepatic view of the inferior vena cava might be useful [50,51,52], but studies on this population of pregnant women are currently lacking. The present study has several limitations. First, we did not perform an analysis of the duration of maternal hypotension. However, as the duration of elective CS from the performance of SA until fetal extraction was mostly limited to less than 10 min, this is unlikely to have biased our results. Moreover, the combination of a continuous monitoring system and the application of a standardized protocol probably limited the duration of each hypotensive episode. Second, we did not consider a more restrictive cut-off of hypotension for pharmacological treatment as this could have been regarded as unethical. Indeed, waiting for a drop of MAP < 55 mmHg could have potentially exposed the newborn to reduced oxygen delivery and ischemia. Third, we did not have a control group treated with an intermittent blood pressure monitoring system. Moreover, we investigated only a selected population of pregnant women (positive PCT) in order to limit the study cohort to the patients that might have benefited more from the hemodynamic monitoring and control of their blood pressure. Fourth, we did not consider the insertion of an arterial catheter in an otherwise healthy population of patients. Finally, our study did not consider the effects of other aspects of the perioperative period that may influence maternal outcomes.

## 5. Conclusions

In a population of pregnant women undergoing elective CS and at risk for perioperative hypotension, we observed a significant prevalence of hypotension according to two different cut-offs using non-invasive continuous hemodynamic monitoring. A protocol that limited pharmacological treatment for maternal hypotension with mean arterial pressure (MAP) values below 60 mmHg did not result in clinically meaningful changes in umbilical pH and Apgar scores. However, neonatal well-being was satisfactory.

## Figures and Tables

**Table 1:** Maternal hypotension according to different cut-offs at seven time points (T0–T6).

Worst Recorded MAP	MAP ≥ 65 mmHg	MAP < 65 mmHg but ≥60 mmHg	MAP < 60 mmHg and Vasopressor Administration
T_0_ n°	140	5 (3.4%)	0
T_1_ n°	140	4 (2.8%)	1 (0.7%)
T_2_ n°	144	0	1 (0.7%)
T_3_ n°	107	22 (15.7%)	16 (11%)
T_4_ n°	74	49 (33.8%)	22 (15.2%)
T_5_ n°	89	38 (26.2%)	18 (12.4%)
T_6_ n°	125	17 (11.7%)	3 (2.1%)
Prevalence of at least one episode of hypotension (%)		54.48%	42.07%

**Table 2:** Etilefrine administration and doses at seven time points. The number of patients treated with etilefrine and the relative doses are presented.

	T_0_	T_1_	T_2_	T_3_	T_4_	T_5_	T_6_	Total
2 mg	0	1	1	8	16	16	3	45
3 mg				3	3	1		7
4 mg					2	1		3
5 mg				5	1			6

**Table 3:** Apgar scores at 1 and 5 min and the frequencies observed.

Apgar Score	1 min	5 min	Frequency % 1 min	Frequency % 5 min
7	2	0	1.4	0
8	4	0	2.8	0
9	74	4	51	2.8
10	65	141	44.9	97.2

## Data Availability

All research data are available from the corresponding author upon reasonable request.

## Supplementary Materials

The following supporting information can be downloaded at: https://www.mdpi.com/article/10.3390/jcm12237295/s1.
