# Supplementary material for: Non-Invasive Monitoring during Caesarean Delivery: Prevalence of Hypotension and Impact on the Newborn
Source: J Clin Med. 2023 Nov 24;12(23):7295. doi: 10.3390/jcm12237295 (PMC10707670; doi:10.3390/jcm12237295)
Supplement: Supplementary file 1 [file jcm-12-07295-s001.zip › jcm-2634407-supplementary.pdf]

# Supplementary Material

**Table S1**. Hemodynamic variables at seven time-points

| | $T_0$ | $T_1$ | $T_2$ | $T_3$ | $T_4$ | $T_5$ | $T_6$ |
|---|---|---|---|---|---|---|---|
| **MAP (mmHg)** | 95±13 | 96±14 | 97±13 | 86±17 | 77±19 | 80±18 | 83±16 |
| **HR b/min** | 98±14.8 | 95±14 | 97±14 | 99±18 | 88±20 | 85±19 | 87±16 |
| **CI L/min/m²** | 4.5±0.8 | 4.6±0.9 | 4.7±0.9 | 4.5±1 | 3.8±0.9 | 3.8±0.8 | 4.2±1 |
| **SVI ml/min/m²** | 46±8 | 48±9 | 49±9 | 46±10 | 43±11 | 46±10.5 | 48±10 |

**Supplementary Material S1.** Hemodynamic variables at seven time-points. Mean arterial pressure (MAP), heart rate (HR), stroke volume index (SVI), Cardiac index (CI) trend observed.

**Figure S1**. Informed consent.

AZIENDA OSPEDALIERO UNIVERSITARIA
"POLICLINICO- VITTORIO EMANUELE" CATANIA
PRESIDIO "GASPARE RODOLICO"
U.O. DI ANESTESIA, RIANIMAZIONE E TERAPIA INTENSIVA
Direttore Prof.ssa Marinella Astuto

## FOGLIO INFORMATIVO
## PER LA PARTECIPAZIONE ALLO STUDIO "MONITORAGGIO EMODINAMICO IN OSTETRICIA"

**Struttura interessata:**
U.O. Anestesia, Rianimazione e Terapia Intensiva
AOU Policlinico - Vittorio Emanuele
Presidio "Gaspare Rodolico"
Via Santa Sofia 78, Catania

**Sperimentatore Responsabile:**
Prof.ssa Marinella Astuto
Direttore U.O. Anestesia, Rianimazione e Terapia Intensiva
AOU Policlinico - Vittorio Emanuele
Via Santa Sofia 78, Catania
Tel. 095 3781117
e-mail: marinella.astuto@gmail.com

Gentile Signora, presso l'Unità Operativa di Anestesia, Rianimazione e Terapia Intensiva dell'Azienda Ospedaliero-Universitaria Policlinico - Vittorio Emanuele di Catania intendiamo svolgere una ricerca osservazionale, quindi che non prevede procedure diverse da quelle già in uso nella normale pratica clinica, che si propone di ottenere informazioni sulle variazioni emodinamiche delle pazienti sottoposte a Taglio Cesareo in anestesia spinale.

Per svolgere questa ricerca avremmo bisogno del suo consenso per poter utilizzare i dati che la riguardano. Prima di prendere la decisione di accettare o rifiutare la preghiamo di leggere con attenzione quanto segue e di chiederci chiarimenti qualora non siano chiare le nostre spiegazioni.

**Vogliamo informarla che se decide di non partecipare alla ricerca che le stiamo proponendo riceverà comunque tutte le cure previste, ed i medici continueranno a seguirla con la massima attenzione assistenziale.**

Per facilitare la sua decisione riassumiamo, nei punti seguenti, le informazioni che crediamo la possano riguardare direttamente e le possano dunque essere utili nella decisione.

Versione 1.0
Data 31 Ottobre 2017

- ***PERCHÉ LE PROPONIAMO DI PARTECIPARE A QUESTO STUDIO***

La ricerca che intendiamo svolgere coinvolgerà le partorienti che, come lei, devono essere sottoposte a taglio cesareo in anestesia spinale e potrebbero sviluppare ipotensione post anestesia sub-aracnoidea.

- ***CHE COSA SI PROPONE QUESTO STUDIO***

Lo studio si propone di raccogliere dati clinici sulle variazioni emodinamiche, prima durante e dopo la somministrazione di un'anestesia sub-aracnoidea, attraverso una fascetta fornita di sensore che viene posizionata su un dito della mano. Lo studio pertanto non prevede che lei si sottoponga a nessuna nuova visita o esame diagnostico aggiuntivo ed è pertanto assolutamente privo di rischi.

- ***INFORMAZIONI CIRCA I RISULTATI DELLO STUDIO***

A studio concluso se lei è interessata e lo richiede le saranno comunicati i risultati dello studio.

- Chi si può contattare in caso di dubbi o domande

Nonostante lo studio non comporti rischi per la sua salute o indagini aggiuntive la invitiamo a contattare il personale di seguito indicato qualora sorgessero eventuali dubbi o domande relative alla ricerca che le è stata proposta. La stessa cosa vale se, in futuro, lei cambiasse idea e decidesse di ritirare il consenso alla partecipazione allo studio.

- Prof.ssa Marinella Astuto mail: marinella.astuto@gmail.com tel 0953781117
- Dr. Paolo Murabito mail: paolomurabito@tiscali.it tel 3293412022
- Dr. Mirko Mineri mail: mirkomineri@gmail.com tel 3926933870

**In conclusione è necessario sottolineare che il protocollo sperimentale è stato redatto in conformità alle Norme di Buona Pratica Clinica dell'Unione Europea e alla Dichiarazione di Helsinki ed è stato approvato dal Comitato Etico dell'Azienda Ospedaliero-Universitaria Policlinico-Vittorio Emanuele.**

***DICHIARAZIONE DI CONSENSO***

Io sottoscritta………………………………………………… dichiaro di aver ricevuto dal Dottor……………………………… in data ……………… spiegazioni esaurienti in merito alla richiesta di partecipazione allo studio "Monitoraggio emodinamico in Ostetricia", secondo quanto riportato nel foglio informativo qui allegato, copia del quale mi è stata consegnata in data …………………….
Dichiaro di aver potuto discutere tali spiegazioni, di aver avuto modo di porre tutte le domande che ho ritenuto necessarie e di aver ricevuto in merito risposte soddisfacenti.
Accetto dunque liberamente di partecipare allo studio proposto.

Data………………

Firma del paziente……...............................................

Data………………..

Firma del medico che ha informato il paziente……………………………….…

AZIENDA OSPEDALIERO UNIVERSITARIA
"POLICLINICO- VITTORIO EMANUELE" CATANIA
PRESIDIO "GASPARE RODOLICO"
U.O. DI ANESTESIA, RIANIMAZIONE E TERAPIA INTENSIVA
Direttore Prof.ssa Marinella Astuto

**Informativa e manifestazione del consenso al trattamento dei dati personali**

**Titolari del trattamento e relative finalità**
Il Centro di sperimentazione, U.O. Anestesia, Rianimazione e Terapia Intensiva, in accordo alle responsabilità previste dalle norme della buona pratica clinica (decreto-legge n. 211/2003), tratterà i Suoi dati personali, in particolare quelli relativi alla sua salute, esclusivamente in funzione della realizzazione dello studio.
Il trattamento dei suoi dati personali è indispensabile allo svolgimento dello studio: il rifiuto di conferirli non Le consentira' di parteciparvi.

**Natura dei dati**
Il medico che La seguirà nello studio La identificherà con un codice: i dati che La riguardano raccolti nel corso dello studio, ad eccezione del Suo nominativo, saranno registrati, elaborati e conservati unitamente a tale codice, alla Sua data di nascita, al sesso, al Suo peso e alla Sua statura. Soltanto il medico e i soggetti autorizzati potranno collegare questo codice al Suo nominativo.

**Modalità del trattamento**
I dati, trattati mediante strumenti anche elettronici, saranno diffusi solo in forma rigorosamente anonima. La diffusione potrà avvenire, ad esempio, attraverso pubblicazioni scientifiche, statistiche e convegni scientifici. La Sua partecipazione allo studio implica che, in conformità alla normativa sulle sperimentazioni cliniche dei medicinali, il personale incaricato del monitoraggio e della verifica dello studio, il Comitato Etico e le autorità sanitarie italiane e straniere potranno conoscere i dati che La riguardano, contenuti anche nella Sua documentazione clinica originale, con modalità tali da garantire la riservatezza della Sua identità.

**Esercizio dei diritti**
Lei potrà inoltre esercitare i diritti di cui all'art. 7 del Codice della Privacy (per es., accedere ai Suoi dati personali, integrarli, aggiornarli, rettificarli, opporsi al loro trattamento per motivi legittimi, ecc.) rivolgendosi direttamente al centro di sperimentazione.
Potrà interrompere in ogni momento e senza fornire alcuna giustificazione la Sua partecipazione allo studio. In tal caso, non saranno inoltre raccolti ulteriori dati che La riguardano, ferma restando l'utilizzazione di quelli eventualmente già raccolti per determinare, senza alterarli, i risultati della ricerca.

**Consenso**
Sottoscrivendo tale modulo acconsento al trattamento dei miei dati personali per gli scopi della ricerca nei limiti e con le modalità indicate nell'informativa fornitami con il presente documento.

Nome e Cognome dell'interessato (in stampatello)......................................................

Firma dell'interessato...................................................................................

Data ....................................................

Versione 1.0
Data 31 Ottobre 2017
